# Journal clubs in Australian medical schools: prevalence, application, and educators’ opinions

**DOI:** 10.3352/jeehp.2020.17.9

**Published:** 2020-02-26

**Authors:** Damian James Ianno, Kelly Mirowska-Allen, Stephen Anthony Kunz, Richard O’Brien

**Affiliations:** 1 Department of Emergency Medicine, Austin Health, Melbourne, Australia; 2 Department of Obstetrics and Gynaecology, Mercy Hospital for Women, Melbourne, Australia; 3 Department of Surgery, Austin Health, Melbourne, Australia; 4 Department of Medicine, Austin Health, Melbourne, Australia; Hallym University, Korea

**Keywords:** Australia, Continuing medical education, Curriculum, Evidence-based medicine, Journal club

## Abstract

**Purpose:**

Medically-focused journal clubs have been used as an educational tool for over 100 years, with research indicating that they improve knowledge, reading behaviour, and critical appraisal skills. However, it is unknown how widespread they are in Australian medical schools, nor the opinions of medical education leaders as to their value.

**Methods:**

A nationwide cross-sectional study was performed among academic leaders from every Australian medical school. Individuals were asked to complete a survey detailing their attitudes towards journal clubs using single- or multiple-answer questions, Likert scales, and ranked data. They were asked whether students at their institutions were able to partake in journal clubs, and if so, provided details on their implementation.

**Results:**

At least 1 response was collected from 18 of 19 Australian medical schools. The response rate was 40.8% (60 of 147), and 36 responses (60.0%) were from heads of clinical schools. Respondents from 15 of 18 institutions (83.3%) stated that their institution had a journal club. Of these, 23 (65.7%) were metropolitan institutions and 12 (34.3%) were rural institutions. Eighteen (51.4%) journal clubs were clinician-led, 13 (37.1%) were run through specific hospital departments, and 23 (65.7%) occurred during clinical years. Most respondents (20 [57.1%]) stated that the primary aim of the journal club was to develop critical appraisal skills.

**Conclusion:**

Journal clubs are a highly regarded educational tool in the armoury of medical school educators, with significant heterogeneity in their structure, geographic prevalence, and intended purpose. Further studies of their efficacy in teaching evidence-based medicine is warranted.

## Introduction

In Australia, the last 2 decades have seen a profound shift in the curriculum and pedagogy of medical education, with less emphasis on traditional lecture-based learning and more emphasis on teaching that can be applied and practiced, such as problem-based learning, simulation, and inter-professional teamwork [1]. The move from a didactic to a collaborative education model has been driven by the rise in graduate medical programs, which necessitated the incorporation of adult learning principles [2]. Another profound change, affecting healthcare worldwide, is that evidence-based medicine has become the gold standard in clinical practice. Medical practitioners have mostly embraced this trend, expanding their roles as healers and teachers to include researchers. The strong focus on research within Australian medicine has had implications for job security, and admission to specialty colleges effectively relies on research production. This trend has not gone unnoticed amongst medical teaching institutions, with many courses now including a research component in their already overloaded curriculum. Partially related to these changes is the fact that more research is now being produced than ever before [3]. The already impossible task of keeping up to date with all relevant, newly published articles is made more so by articles whose content is often incomplete, incorrect, or misleading, which may explain—at least in part—why a large proportion of medical research cannot be reproduced [4]. Therefore, the ability to critically appraise research articles in an effective manner has never been more important. It is this evolution of medicine that makes journal clubs, like bedside tutorials and human dissection, one of the few medical education tools to withstand the test of time. Despite this, our knowledge of journal clubs in Australian medical schools is lacking. Specifically, we do not know the prevalence of journal clubs, how they are implemented, or the opinions of academics towards their use as an educational tool. Our study aims to address these questions.

## Methods

### Ethics statement

Approval for this study was obtained from the University of Melbourne Department of Medical Education Human Ethics Advisory Group (Ethics ID: 1648448.1).

All procedures followed were in accordance with the ethical standards of the responsible committee on human experimentation (institutional and national) and with the Helsinki Declaration of 1964 and its later amendments. Informed consent was obtained from all survey respondents included in the study and a plain-language statement was provided. This study was designed to improve quality in medical education through expert opinion in the form of a survey. Participants were made aware of the study and no staff—excluding the authors listed—had access to the data. Confidentiality was maintained at all times. The study did not involve any identifying data that could breach privacy. Respondents completed the survey on a voluntary basis, after which the results were pooled and analysed.

### Study design

A cross-sectional survey research design was used.

### Materials and/or subjects

We conducted a survey of key academics at all Australian medical schools to identify whether (1) their school had a journal club, (2) how the journal club was implemented if there was one, and (3) what they personally thought of journal clubs as an educational tool. The survey was administered online using the survey tool provided by Survey Monkey (www.surveymonkey.com/). The complete survey is available as Supplement 1.

Australian medical schools were identified by visiting the websites of Medical Deans Australia and New Zealand and the Australian Medical Students Association, both of which provide a list of current institutions. We then manually searched the websites of these universities for the names and contact details of medical and clinical school heads and deputy heads. For universities who employed multiple deputy heads of school, each of whom focused on a different department, we selected the role most likely to be involved with a journal club (e.g., the deputy head of research). If the necessary information could not be gleaned from online searches, the university was contacted directly.

Included in our survey were all Australian medical schools that contained a cohort of students at every year level. This resulted in the exclusion of 2 newly established medical schools. All clinical schools based in Australia were included in the study, with the exception of schools consisting solely of a rural general practice clinic.

### Technical information

A 10-point survey was developed to gain information on the participants’ roles within the university, preferred journal club logistics, attitudes towards journal clubs, and whether or not opportunities were available for medical students to partake in them. If a participant indicated that they were aware of a journal club’s existence at their institution, we asked 7 more questions pertaining to its implementation. For the purposes of our study we defined a journal club as “a group of people, including medical students, who meet periodically to discuss research articles in the scientific or medical field. Discussion may include, but is not limited to clinical application, biostatistics, epidemiology, and critical evaluation.” The survey, along with a plain-language statement, was initially delivered online via email, with 2 reminders. If no response was received electronically, the survey was delivered through standard mail. Responses were collected and securely stored on an Excel spreadsheet used for qualitative analysis.

The respondents were dichotomised according to the Australian Standard Geographical Classification–Remoteness Area [5]. The classification ranges from RA1 (major cities of Australia) to RA5 (very remote Australia). All respondents residing in RA1 areas were considered ‘metropolitan,’ with the remainder considered as being ‘rural.’

### Statistical analysis

Responses were collected using single- or multiple-answer questions, 10-point Likert scales, and ranked data.

A ranking average was used to determine the intended purpose of journal clubs in medical education. The ranking average was determined as follows: w=weight of ranked position and x=response count for answer choice {(x1w1+x2w2 ... xnwn)÷(total)}. The primary intended purpose of journal clubs, which respondents ranked as the most important, had the largest weight. The lowest-rated intended purpose (ranked in the last position) had a weight of 1. The criteria with the highest average score were deemed the primary purpose of journal clubs.

## Results

One or more responses were collected from 18 of the 19 Australian medical schools (94.7%). This corresponded to a total of 60 of 147 (40.8%) responses, and most respondents (36 [60%]) were clinical school heads. Deputy heads of medical schools and academic leaders each comprised 7 (11.7%) responses, while heads of medical schools accounted for 6 (10.0%), and deputy head of clinical schools made up 4 (6.7%). Of the 60 collected responses, 35 (58.3%) were educators based in metropolitan centres, with the remaining 25 (41.7%) based in rural centres.

### Opinions of educators

Students’ ability to critically evaluate evidence was rated of great importance by these academics (median, 9; interquartile range [IQR], 8–10), as was an appreciation of research methods (median, 8; IQR, 7–9), and an understanding of biostatistics and epidemiology (median, 7; IQR, 6–8).

Generally speaking, journal clubs were highly regarded for the education of medical students (median, 7; IQR, 6–8) and were thought to be effective at teaching research skills (median, 7; IQR, 6–8).

When asked to rank the purpose of a journal club on a scale from 1 to 6 (with 1 indicating the highest importance) as demonstrated in Table 1, the development of critical appraisal skills was deemed to be the most important purpose (1), followed by a forum for the discussion and debate of medical topics using evidence (2), a means of encouraging an appreciation of research (3), a way to disseminate information relating to good practice (4), a method of keeping students abreast of new research (5), and a method of teaching biostatistics and epidemiology (6).

Twenty-one (35.0%) respondents felt that journal clubs were of such importance that attendance should be made compulsory. However, 20 (33.3%) felt that journal club attendance should not be mandatory, while 19 (31.7%) were unsure.

The plurality of respondents (25 [41.7%]) thought that journal clubs should only be run during the clinical years, 25 (41.7%). This was followed closely by the opinion that they should be run throughout medical school (23 [38.3%]), and less popular options were that journal clubs should be held during a compulsory research term (5 [8.3%]) and during the final year only (5 [8.3%]). Two responders (3.3%) felt that journal clubs should never be implemented during medical school.

### Prevalence and implementation

There was a high prevalence of journal clubs among medical schools, with 15 of 18 universities (83.3%) stating that they incorporated a journal club in their curriculum. Of the 60 respondents to the survey, 35 (58.3%) stated that their clinical school or institution had a journal club, and they were asked a further series of questions. Twenty-three (65.7%) of these respondents were based at metropolitan sites, while the remaining 12 (34.3%) were rurally based. Only 48.0% (12 of 25) of rurally based institutions had a journal club, compared to 65.7% (23 of 35) of institutions at metropolitan sites.

As seen in Table 2, journal clubs were most frequently described as clinician-led (18 [51.4%]), student-led (11 [31.4%]), or a combination thereof (5 [14.3%]). One journal club (2.9%) was described as being led through the hospital administration. The department or organization responsible for running the journal club was most frequently a specific specialty department (13 [37.1%]), followed by the clinical school (10 [28.6%]), and in fewer cases directly by the university (9 [25.7%]) or student associations (2 [5.7%]). One respondent (2.9%) reported a combination of these.

Journal clubs were most commonly conducted during the clinical school years (23 [65.7%]), with fewer being available throughout medical school (4 [11.4%]), the pre-clinical years (4 [11.4%]), and the final year (4 [11.4%]). Two respondents (5.7%) stated that the journal club was held during a compulsory research term, and 2 (5.7%) were unsure when their journal club was run. As responders could select more than 1 time-point, our survey revealed multiple opportunities for students, at varying year levels, to gain exposure to a journal club.

Journal clubs most commonly met either weekly (11 [31.4%]) or monthly (11 [31.4%]). Fortnightly gatherings were less common (6 [17.1%]), and some respondents reported “other” frequencies such as once every 2 months (1 [2.9%]), variably (3 [8.6%]), or unsure (3 [8.6%]). Journal clubs were reported as mandatory in 11 (31.4%) cases, while 21 (60.0%) were voluntary and 3 (8.6%) respondents indicated that they were unsure.

The aims of existing journal clubs were to develop critical appraisal skills (20 [57.1%]), to provide a forum to discuss and debate medical topics using evidence (8 [22.9%]), to provide a means of disseminating information relating to good practice (3 [8.6%]), to encourage an appreciation of research (2 [5.7%]), and to keep students abreast of new research (1 [2.9%]). One person (2.9%) felt that the aims of a journal club differed depending on whether they were faculty or student-led, with the former being more focused on the teaching of critical appraisal, and the latter on fostering an appreciation of research.

## Discussion

Our study demonstrated that journal clubs are popular in Australian medical schools, with over 4 in 5 of the surveyed universities implementing a journal club for the teaching of their students. Although no study has previously investigated the prevalence of journal clubs in this specific medical student cohort, the rate found in our study is similar to that reported for medical residency programs [6,7].

It is hardly surprising that the majority of journal clubs were found in metropolitan sites, and the reasons for this discrepancy are likely multifactorial. Firstly, rurally-based clinical schools contain fewer students than their metropolitan counterparts [8]. Given that the establishment of a journal club and the facilitation of discussions could be thought to require a ‘critical mass’ of participants in order to be meaningful, implementing a journal club at rural institutions may not have been as feasible as at metropolitan sites. Sidorov [6] demonstrated that the strongest determinant of the success of journal clubs—as defined by having high attendance or long, continuous existence—was having smaller groups. However, other reviews have shown that the number of participants did not positively or negatively impact the overall success of a journal club [9], indicating that although journal clubs are less popular rurally, they will not be less efficacious if implemented.

Furthermore, universities with a research focus—especially those affiliated with dedicated research institutions—are largely metropolitan, and therefore may place greater importance on journal clubs than less research-affiliated regional centres. Metropolitan sites are also more likely to offer specialty-based journal clubs given the greater number of specialty departments (e.g., cardiology). Whatever the reason for the disparity, it is unlikely to have a significant effect on the academic performance of Australian rural students, given that they have demonstrated at least equivalence to their metropolitan counterparts [10].

Our study found that the critical appraisal of evidence was the principal goal and the primary purpose of existing journal clubs. This is in keeping with previous research, including a seminal study of Linzer et al. [11] and other studies and reviews of journal clubs [12], where the primary goal was found to be teaching of critical appraisal skills. Given the recent focus on evidence-based medicine both abroad [13,14] and domestically [15], the importance of critical appraisal skills is well-founded and demonstrates the evolution of journal clubs from a convenient way to share medical discoveries to one that is arguably essential in the preparation of medical students for practice.

With regards to implementation, it was not surprising that journal clubs were predominantly clinician-led, as most medical students will require external input to decipher the content of journal articles. However, the popularity of student- or peer-led teaching activities has been growing since the 1990s, particularly in the medical field, as has the body of research exploring its benefits and limitations [16,17]. While no study has investigated this method in the setting of a medical student journal club, several studies have examined the efficacy of peer-teaching in clinical examination skills, problem-based learning, and examination revision. In their systematic review, Yu et al. [16] found that peer teaching appeared to be equivalent to conventional faculty-led teaching in a selective context, likely due to the cognitive and social congruence between the student-teacher and their peers.

Most journal clubs were held during the clinical years, which was also identified as being the ideal time during medical school by respondents. This was not a surprising finding given that students require a baseline level of knowledge achieved in their pre-clinical education years to understand the content of the articles reviewed. Additionally, when delivered during the clinical years, students would be able to apply knowledge obtained from journal articles to clinical interactions in the hospital and to patient care.

The finding that journal clubs usually met weekly and fortnightly confirms the conclusions of a previous systematic review that weekly meetings were the most common, followed by monthly and fortnightly [9].

While some journal clubs made student attendance mandatory, the majority did not. This difference could reflect both institutional culture and university requirements, with some clinical schools providing students the ability to attend opportunistically for furthering personal learning, while others may have mandated attendance as a ‘hurdle’ requirement in the curriculum. A systematic review by Deenadayalan et al. [18] in 2008 investigating the key factors of running an effective journal club found that making attendance mandatory was important for ensuring ongoing success. It is therefore unsurprising that in in a review of Harris et al. [9] in 2011 on journal club structure, the majority of studies in which attendance was specified required it to be mandatory.

### Limitations

Despite a seemingly low response rate from eligible respondents, the survey was designed in way that accounted for non-responders, with in-built redundancy for each site. The response rate was also slightly higher than the expected response rate of 35% for web surveys [19].

While under-sampling of academics was not an issue, it is possible that a small number of journal clubs— especially those that were led by students or allied health professionals—may have been missed. Similarly, fewer respondents were sourced per site regionally than in metropolitan areas. This again may have resulted in under-reporting. Finally, certain points that were emphasised in other reviews of journal clubs were not included in our survey, including the number of participants per session, the longevity of journal clubs, and the presence of external sponsorship.

### Conclusions

This nationwide study—the first of its kind—emphasizes the prevalence of journal clubs in Australian medical schools. Academics within these institutions believed that journal clubs are beneficial for the teaching of students, particularly with respect to critical appraisal skills. As research articles are being produced and disseminated at an exponential rate, teaching our future doctors the skills required to appropriately evaluate scientific articles is crucial for developing a generation of clinicians adept in providing evidence-based medicine. Our study provides a unique understanding of medical education leaders’ perceptions regarding the utility of journal clubs, and provides a framework of expert insights for the development of future journal clubs.

## Figures and Tables

**Table 1.** Educators’ ranking of the intended purpose of journal clubs in medical education

Criteria	Weighted scores^a)^	Rank
Critical appraisal skills development	4.88	1
A forum to discuss and debate medical topics using evidence	3.93	2
Encourage an appreciation of research	3.42	3
A forum to disseminate information relating to good practice	3.25	4
Keeping students abreast of new research	2.82	5
Teach biostatistics and epidemiology	2.68	6

Responders were asked ‘What do you think is the purpose of a journal club for medical school students?’ and ranked criteria from most important (1) to least important (6) (n=60).

a) Calculated weighted rank scores.

**Table 2.** Implementation and opinions of medical educators from institutions with journal clubs

Survey question and response	Frequency (%)
Who leads the journal club?^a)^	
Clinician-led	18 (51.4)
Student-led	11 (31.4)
Both	5 (14.3)
Other (hospital administration)	1 (2.9)
Who runs the journal club?^a)^	
Specialty specific departments	13 (37.1)
Clinical school	10 (28.6)
University	9 (25.7)
Student associations	2 (5.7)
Other (combination of clinical school and university)	1 (2.9)
How often does journal club meet?	
Weekly	11 (31.4)
Monthly	11 (31.4)
Fortnightly	6 (17.1)
Variably	3 (8.6)
Unsure	3 (8.6)
Bimonthly	1 (2.9)
Location of journal club	
Metro	23 (65.7)
Rural	12 (34.3)
What is the primary aim of your journal club?^a)^	
Critical appraisal skills development	20 (57.1)
A forum to discuss and debate medical topics using evidence	8 (22.9)
A forum to disseminate information relating to good practice	3 (8.6)
Encourage an appreciation of research	2 (5.7)
Keeping students abreast of new research	1 (2.9)
Other	1 (2.9)
Teach biostatistics and epidemiology	0
Is journal club mandatory?	
Mandatory	11 (31.4)
Unsure	3 (8.6)
Not mandatory	21 (60.0)

Results of the medical educator survey for educators with journal clubs (n=35).

a) Total of percentages is less than/greater than 100% due to rounding.

## Supplementary materials

Data files are available from Harvard Dataverse: https://doi.org/10.7910/DVN/KEJKEY

Supplement 1. Questionnaire used.

Supplement 2. Audio recording of the abstract.
