# Supplementary material for: Journal clubs in Australian medical schools: prevalence, application, and educators’ opinions
Source: J Educ Eval Health Prof. 2020 Feb 26;17:9. doi: 10.3352/jeehp.2020.17.9 (PMC7365995; doi:10.3352/jeehp.2020.17.9)
Supplement: Supplementary file 2 — Supplement 1. Questionnaire used. [file jeehp-17-09-suppl.pdf]

## 1. Background information

* 1. I am a:

* 2. To which medical school do you belong:

3. If applicable, to which clinical school do you belong?

* 4. How valuable do you think a journal club is to the education of medical students?

| 1 - Not valuable | 2 | 3 | 4 | 5 | 6 | 7 | 8 | 9 | 10 - Extremely valuable |
|---|---|---|---|---|---|---|---|---|---|
| ○ | ○ | ○ | ○ | ○ | ○ | ○ | ○ | ○ | ○ |

5. How effective do you think a journal club is at teaching research skills?

| 1 - Not valuable | 2 | 3 | 4 | 5 | 6 | 7 | 8 | 9 | 10 - Extremely valuable |
|---|---|---|---|---|---|---|---|---|---|
| ○ | ○ | ○ | ○ | ○ | ○ | ○ | ○ | ○ | ○ |

* 6. Should journal club attendance be compulsory for medical students?

- ○ Yes
- ○ Maybe
- ○ No

* 7. To the best of your knowledge do your medical students partake in a journal club?

- ○ Yes
- ○ No
- ○ Unsure

## 2. Your medical schools journal club

* 8. Is the journal club:

- ◯ Student lead
- ◯ Clinician lead
- ◯ Other (please specify)

* 9. At what time during the medical school course do students partake?*(You may select more than one option if applicable)*

- ☐ Throughout medical school
- ☐ In the pre-clinical years
- ☐ During the clinical years
- ☐ During a compulsory research term
- ☐ During final year
- ☐ Other (please specify)

* 10. At what time during the medical school course should students partake?

- ◯ Throughout medical school
- ◯ In the pre-clinical years
- ◯ During the clinical years
- ◯ During a compulsory research term
- ◯ During final year
- ◯ Never

* 11. Who runs the journal club?

- University
- Student association
- Clinical School
- Specific hospital department(s)
- Other (please specify)

* 12. Is the journal club mandatory?

- Yes
- Unsure
- No

* 13. What is the primary aim of your journal club? *(Please select one answer)*

- My medical school does not have a journal club
- Develop critical appraisal skills
- Encouraging an appreciation of research
- Keeping students abreast of new research
- Teaching students biostatistics and epidemiology
- A forum to discuss and debate medical topics using evidence
- A forum to disseminate information relating to good practice
- Other (please specify)

14. What do you think is the purpose of a Journal Club for medical school students?*(You may select more then one answer)*

- Critical appraisal skills development
- Encourage an appreciation of research
- Keep students abreast of new research
- Teach biostatistics and epidemiology
- A way to disseminate information relating to good practice
- A way to encourage discussion and debate of medical topics using evidence

15. How important is it that the following research skills are attained by students during their medical degree?

| | Not at all 1 | 2 | 3 | 4 | 5 | 6 | 7 | 8 | 9 | Very much 10 |
|---|---|---|---|---|---|---|---|---|---|---|
| Critical evaluation of evidence and research | ○ | ○ | ○ | ○ | ○ | ○ | ○ | ○ | ○ | ○ |
| Understanding of biostatistics and epidemiology | ○ | ○ | ○ | ○ | ○ | ○ | ○ | ○ | ○ | ○ |
| Understanding the research method | ○ | ○ | ○ | ○ | ○ | ○ | ○ | ○ | ○ | ○ |

16. Do you have any other comments you would like to make regarding the use of journal clubs as a learning tool in a medical course?
